# Kinetics and Characterization of Degradation Products of Dihydralazine and Hydrochlorothiazide in Binary Mixture by HPLC-UV, LC-DAD and LC–MS Methods

**DOI:** 10.1007/s10337-018-3555-8

**Published:** 2018-06-25

**Authors:** Anna Gumieniczek, Justyna Galeza, Tomasz Mroczek, Krzysztof Wojtanowski, Katarzyna Lipska, Rafał Pietras

**Affiliations:** 1 Department of Medicinal Chemistry, Medical University of Lublin, Jaczewskiego 4, 20-090 Lublin, Poland; 2 Department of Pharmacognosy with Medicinal Plant Unit, Medical University of Lublin, Chodźki 1, 20-093 Lublin, Poland

**Keywords:** Dihydralazine and hydrochlorothiazide, Kinetics of degradation, Degradation products, Interactions, HPLC-UV, LC-DAD and LC–MS methods

## Abstract

Dihydralazine and hydrochlorothiazide were stored at high temperature and humidity, under UV/Vis light and different pH, as individual drugs and the mixture. Then, a sensitive and selective HPLC-UV method was developed for simultaneous determination of dihydralazine and hydrochlorothiazide in presence of their degradation products. Finally, the degradation products were characterized through LC-DAD and LC–MS methods. Dihydralazine was sensitive to high temperature and humidity, UV/Vis light and pH ≥ 7. At the same time, it was resistant to acidic conditions. Hydrochlorothiazide was sensitive to high temperature and humidity, UV/Vis light and changes in pH. Its highest level of degradation was observed in 1 M HCl. Degradation of the drugs was higher when they were stressed in the mixture. In the case of dihydralazine, the percentage degradation was 5–15 times higher. What is more, dihydralazine became sensitive to acidic conditions. Hydrochlorothiazide was shown to be more sensitive to UV/Vis light and pH > 4. Degradation of dihydralazine and hydrochlorothiazide followed first-order kinetics. The quickest degradation of dihydralazine was found to be in 1 M NaOH while of hydrochlorothiazide was in 1 M HCl (individual hydrochlorothiazide) or at pH 7–10 (hydrochlorothiazide in the mixture). A number of new degradation products were detected and some of them were identified by our LC-DAD and LC–MS methods. In the stressed individual samples, (phenylmethyl)hydrazine and 1,2,4-benzothiadiazine-7-sulfonamide 1,1-dioxide were observed for the first time. Interactions between dihydralazine and hydrochlorothiazide in the mixture were confirmed by additional degradation products, e.g., 2H-1,2,4-benzothiadiazine-7-sulfonamide 1,1,4-trioxide.

## Introduction

Hydralazine and its derivative dihydralazine are used in the treatment of hypertension in Europe, the USA and China [1]. Because of their special action in some types of hypertension, they are included in European Pharmacopoeia (Eur. Ph.) [2]. They both are arterial vasodilators that can reduce the resistance in arterial vessels. Because of this unique mechanism of action, they can be combined with other drugs of complementary effects, e.g., with hydrochlorothiazide from diuretics. Two-component formulations of hydralazine and hydrochlorothiazide are already present in the USA and China.

Although two-component tablets present many advantages, they introduce the problem of interactions between the active ingredients. For example, degradation of one drug may be accelerated by another one. Interactions can also lead to generate new degradation products. Therefore, the main goal of the present study was to examine stability of dihydralazine and hydrochlorothiazide, as individuals and in the mixture, to detect such interactions using HPLC-UV, LC-DAD and LC–MS methods.

Only few chromatographic procedures (HPLC) have been reported so far for determination of dihydralazine alone [3] or in presence of other drugs like hydrochlorothiazide, triamteren and clonidine [4, 5]. In the study of Raul et al. [3], dihydralazine was determined on a C18 column with a mobile phase consisted of phosphate buffer of pH 3.0 and acetonitrile, and UV detection at 305 nm. However, there is no any report concerning forced degradation of dihydralazine. In consequence, kinetics of degradation as well as characterization of degradation products were not reported at all.

More HPLC methods were elaborated for determination of hydrochlorothiazide as an individual analyte [6, 7] or in presence of other drugs like sartans [8–10], angiotensin-converting enzyme inhibitors [11–13], beta blockers [14–16] and calcium channel blockers [17, 18]. Some HPLC methods were described as stability-indicating procedures capable to determine hydrochlorothiazide in presence of its degradation products [19–26].

In the study of Che [4], determination of dihydralazine and hydrochlorothiazide was performed using a CN column, while a mobile phase consisted of acetonitrile and sodium heptane sulphonate. Two different wavelengths were used for UV detection, i.e., 310 and 267 nm, for dihydralazine and hydrochlorothiazide, respectively. Simultaneous determination of dihydralazine and hydrochlorothiazide was elaborated by Jin et al. [5] using gradient elution on a C18 column. Acetonitrile and phosphate buffer of pH 3.0 containing sodium heptane sulphonate were used as mobile phases A and B, respectively. From these reports, it was clearly seen that simultaneous determination of these two drugs presenting different chemical properties, could be a big analytical challenge.

Stability of hydrochlorothiazide in a solid state was tested at high temperature in the range 60–110 °C [19, 20, 22–25, 27]. As far as photostability is concerned, experiments were conducted at one wavelength (254 or 256 nm) [20, 24, 25] or in the entire UV range [24, 28–30]. They were carried out in a solid state [20, 22, 24, 28, 31] and less frequently in solutions [25, 29, 30]. Stability of hydrochlorothiazide was studied in 1M HCl [19–22, 25, 30–32], 5 M HCl [28], 1 M NaOH [19–22, 25, 27, 31–37], 5 M NaOH [28], buffers [23] and methanol [19, 25]. One study on degradation of hydrochlorothiazide by LC/MS method was reported in the literature [27]. However, kinetics of degradation was studied incidentally and only scarce information in this area was found in the literature [23].

Thus, a new quantitative HPLC method for simultaneous determination of dihydralazine and hydrochlorothiazide in presence of their degradation products was elaborated and validated. Then, the concentration of non-degraded dihydralazine and hydrochlorothiazide as a function of degradation time was investigated, to determine kinetics of degradation. The next step was to elucidate the possible degradation pattern of these drugs using LC-DAD and LC–MS methods.

The results reported here can be useful in development of a new combined formulation of dihydralazine and hydrochlorothiazide, by knowing dangerous conditions for both constituents. In addition, these data may be the starting point for further studies on new degradation products in terms of their potential toxicity.

## Experimental

### Materials and Methods

#### Materials

Pharmaceutical grade (Eur. Ph.) dihydralazine sulfate and hydrochlorothiazide, and tetrabutylammonium hydrogen sulfate for analysis from Sigma-Aldrich (St. Louis, MO, USA), ammonium formate, formic acid, acetonitrile and methanol for LC from Merck (Darmstadt, Germany), acetic acid (CH_3_COOH), sodium acetate (CH_3_COONa), hydrochloric acid, sodium chloride (NaCl), sodium tetraborate (Na_2_B_4_O_7_), sulphuric acid, sodium hydrogen phosphate (NaHPO_4_), sodium hydroxide (NaOH), kalium dihydrogen phosphate (KH_2_PO_4_) and kalium hydroxide for analysis from POCh (Gliwice, Poland), acetonitrile and water for LC–MS from J.T. Baker (Center Valley, PA, USA), Dihydralazinum^®^ tablets 25 mg from Pabianickie Zakłady Farmaceutyczne (Pabianice, Poland) and Hydrochlorothiazidum^®^ tablets 25 mg from Polpharma (Starogard Gdanski, Poland) were used. Acetate buffer was prepared with 0.2 M CH_3_COOH and 0.2 M CH_3_COONa. Phosphate buffer was prepared with 0.067 M KH_2_PO_4_ and 0.067 M Na_2_HPO_4_. Borate buffer was prepared with 0.05 M Na_2_B_4_O_7_ and 0.1 M NaOH. Buffers were prepared as described in Eur. Ph [2]. Buffers for kinetic studies have the same ionic strength of 1 M which was attained with 4 M NaCl. The pH measurements were done with a pH-meter HI9024C from Hanna Instruments (Padova, Italy).

### HPLC-UV Method

#### Chromatographic Conditions

Analysis was performed with a model 306 pump with a loop Rheodyne (20 µL) and a model UV170 detector controlled by Omnic software from Gilson (Middleton, WI, USA). Separation was carried out on a LiChrospher^®^CN column (125 × 4.0 mm, 5 µm) from Merck. The column was housed in a column heater set at 25 °C. The mobile phase consisted of water, 0.02 M tetrabutylammonium hydrogen sulfate and acetonitrile (20:65:15, v/v/v) adjusted to pH 3.5 with 0.5 M sulphuric acid. The flow rate of the mobile phase was 1.4 ml mL^−1^ while the detection was done at 235 nm.

#### Robustness

Small changes of analytical conditions, i.e., acetonitrile content in a mobile phase (15 ± 2), pH (3.5 ± 0.1 unit), flow rate of the mobile phase (1.4 ± 0.2 ml mL^−1^), detection wavelength (235 ± 3 nm) and column temperature (25 ± 2 °C) were made to study robustness of the developed method. One factor was changed at a time. For each combination, three injections were carried out, using a working solution containing 70 µg mL^−1^ of dihydralazine and hydrochlorothiazide. Finally, robustness of the method was expressed in the forms of asymmetry factors and peak areas.

#### Linearity

Stock solutions of dihydralazine and hydrochlorothiazide (1 mg mL^−1^) were used to obtain the working solutions of the drugs in the range from 20 to 120 µg mL^−1^. Then, six injections were made onto the column for each concentration. The peak areas were plotted against the corresponding concentration of the drugs to construct the calibration equations. The limit of detection (LOD) and the limit of quantification (LOQ) were determined from the standard deviation of the intercepts and slopes of the calibration lines at low concentrations, using 3.3 and 10 factors for LOD and LOQ, respectively.

#### Precision and Accuracy

Precision was determined by analyzing the working solutions containing 30, 70 and 110 µg mL^−1^ of dihydralazine and hydrochlorothiazide, three times during the same day and then on three subsequent days. Accuracy was estimated by determining both active substances in six model mixtures and comparing the determined amounts to the nominal values. The weighed portions of powdered tablets containing 25 mg of dihydralazine and 25 mg hydrochlorothiazide were transferred to 25 ml volumetric flasks with ca. 15 mL of methanol, sonicated for 30 min, diluted to the mark and filtered by nylon membrane filters (0.45 µm). Then, 0.6 mL volumes were diluted to 10 mL and analyzed by the HPLC method described above. The assay was repeated six times, individually weighing the respective tablet powders.

The concentrations of dihydralazine or hydrochlorothiazide were calculated using respective calibration equations and expressed as RSD for precision and percentage recovery for accuracy.

### Degradation in a Solid State

Solid mixture containing dihydralazine and hydrochlorothiazide was prepared by weighing equal amounts of individual substances and mixing them thoroughly in an agate mortar. Individual substances and their mixture were placed in standardized small flat vessels, so that the thickness of the layer was approximately 3 mm. The samples were placed in a climate chamber KBF P240 from Binder (Neckarsulm, Germany) at 70 °C and 80% RH for 2 months. After forced degradation, 10 mg of individual substances or 20 mg of the mixture were weighed and dissolved with methanol to obtain solutions of concentration 1.0 mg mL^−1^. After diluting with methanol to cover the linearity range, the solutions were analyzed by our HPLC-UV method. The procedure was repeated three times for each sample, and the concentrations of dihydralazine or hydrochlorothiazide remaining after degradation were calculated from the linear calibration equations.

### Degradation in a Liquid State

#### Photodegradation

Equal volumes (2 mL) of the stock solutions of dihydralazine or hydrochlorothiazide (4 mg mL^−1^) were dispensed to quartz glass-stoppered dishes (individually stressed drugs). Equal volumes (1 mL) of the stock solutions of dihydralazine or hydrochlorothiazide (8 mg mL^−1^) were mixed in quartz glass-stoppered dishes to obtain the mixtures. The samples were placed in a Suntest CPS Plus chamber from Atlas (Linsengericht, Germany) and exposed to UV/Vis light in the range 300–800 nm, with energy equal to 18,902, 56,706 and 113,412 kJ m^2−1^. After forced degradation, the solutions were diluted with methanol to cover the linearity range and analyzed by our HPLC-UV method. The procedure was repeated three times for each sample, and the concentrations of non-degraded dihydralazine or hydrochlorothiazide were calculated from the linear calibration equations.

#### Kinetics

From the stock solutions of dihydralazine or hydrochlorothiazide (4 mg mL^− 1^), 1 mL volumes were dispensed to small glass tubes from Medlab (Raszyn, Poland) (individually stressed drugs). From the stock solutions of dihydralazine or hydrochlorothiazide (8 mg mL^−1^), 0.5 mL volumes were dispensed in a similar way and mixed together. To each tube, 1 mL of appropriate stressor (1 M HCl, 1 M NaOH, buffers of pH 4, 7 and 10) was added. The tubes were tightly closed with stoppers and placed in a thermostated water bath from WSL (Warszawa, Poland) at 80 °C. The samples were removed from the bath after subsequently 15, 30, 45, 60, 75, 90, 105, 120, 135, 150, 165, 180, 195, 210, 225, 240, 255, 270, 285 and 300 min. They were immediately cooled and neutralized if necessary. After diluting with methanol to covering the linearity range, each sample was analyzed using our HPLC-UV method. The procedure was repeated three times for each sample, and the concentrations of non-degraded dihydralazine or hydrochlorothiazide were calculated from the linear calibration equations.

When the level of degradation was at least 30% during 300 min, kinetic parameters were calculated. The logarithm of the concentration of non-degraded substance was plotted against time of degradation to determine the order of reactions. The equations *y* = *ax* + *b* and *R*^2^ coefficients were obtained from which further kinetic parameters, i.e., degradation rate constant (*k*) and degradation time of 50% substance (*t*_0.5_) were calculated.

### LC-DAD and LC–MS Methods

Before analysis, the buffers were removed from the samples using Bakerbond SPE C8 disposable extraction columns (3 mL) from J.T. Baker and an UCT positive pressure Manifold station (Horsham, PA, USA). The ions were removed from the bed with water, and then substances of interest were eluted with methanol. Respective fractions were pooled and dried under vacuum. Before LC analysis, they were reconstituted with acetonitrile.

The samples were analyzed with a 6530B accurate-mass-QTOF-MS spectrometer with a dual ESI-Jet Stream ion source, using an Eclipse XDB C18 (150 × 4.6 mm, 3.5 µm) column from Agilent Technologies (Santa Clara, CA, USA). The chromatograph was equipped with a DAD, an autosampler, a binary gradient pump, and a column oven. Acetonitrile–water (1:99, v/v) with 10 mM ammonium formate (0.1%) (solvent A) and acetonitrile–water (95:5, v/v) with 10 mM ammonium formate (0.1%) (solvent B) were used as mobile phases. The following elution procedure was adopted: 0–60 min, 0–95% of solvent B with a stable flow rate 0.4 mL min^−1^. The injection volume for the samples was 10 µL. The analysis was conducted at 25 °C.

LC/MS analysis was performed according to the following parameters of the ion source: a negative ion mode (–ESI), gas (N_2_) flow rate 12 L min^−1^, nebulizer pressure 35 psig, vaporizer temperature 350 °C, sheath gas temperature 400 °C, sheath gas (N_2_) flow 12 L min^−1^, *m*/*z* range 100–1000 mass units with an acquisition mode auto MS/MS, collision induced dissociation (CID) 10 and 40 eV with MS scan rate of 1 spectrum per s and 2 spectra per cycle, VCap 4000 V, skimmer 65 V, fragmentor 150 V and Octopole RF Peak 750 V. Additionally, the analysis was made in auto MS/MS with excluded *m*/*z* at 966.0007 and 112.9856 for negative ion mode, corresponding to the *m*/*z* of reference ions.

The obtained experimental mass values were used to generate molecular formula of the products, on which basis their structures were predicted. The most postulated structures were justified through the mechanisms of their formation.

## Results and Discussion

### Development and Validation of LC-UV Method

A simple, isocratic HPLC-UV method was developed for simultaneous determination of dihydralazine and hydrochlorothiazide in presence of their degradation products. It is worth mentioning that similar reports concerning dihydralazine were not found in the literature.

**Fig. 1:**
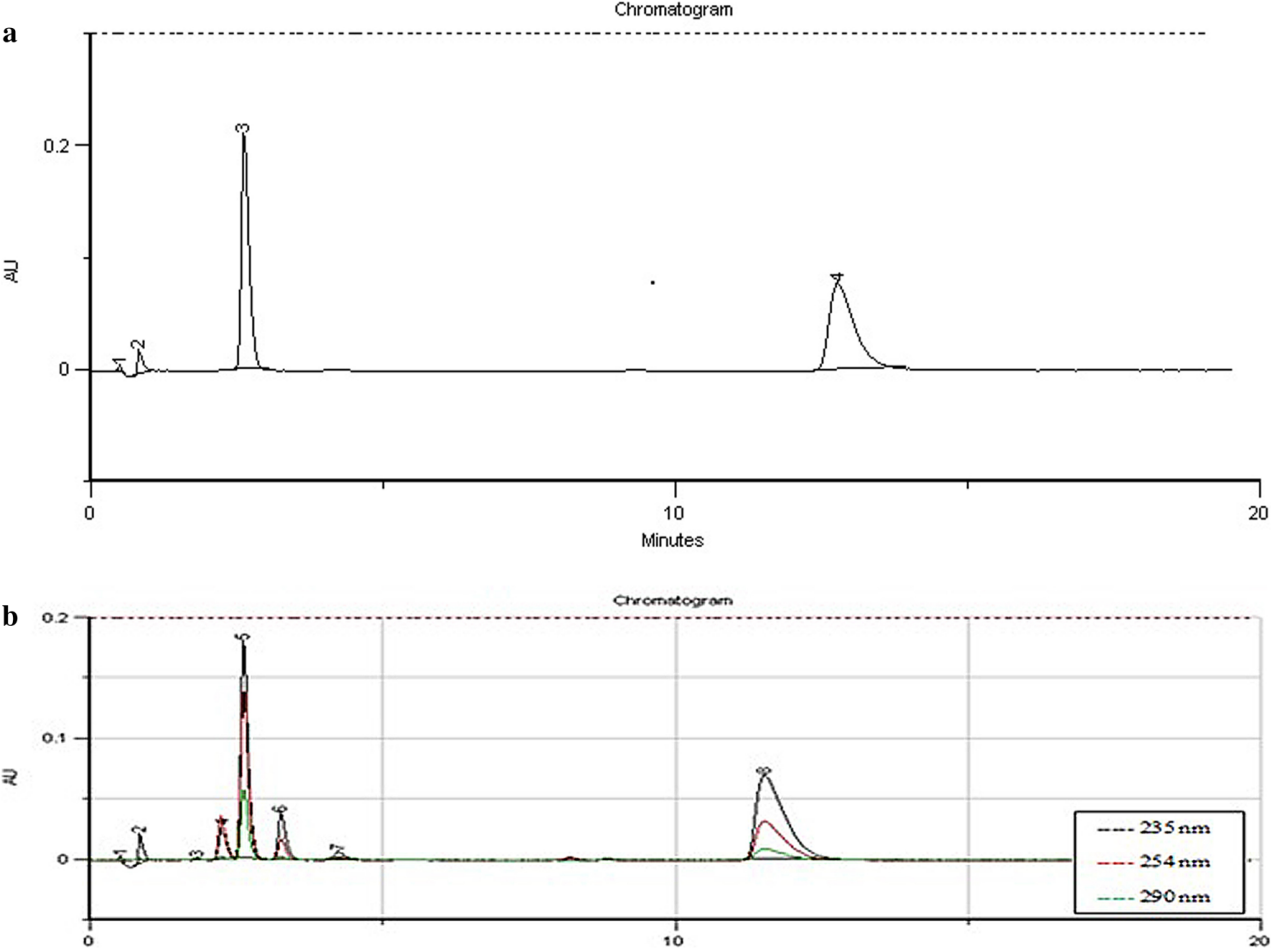
Chromatograms HPLC-UV: **a** dihydralazine (4) and hydrochlorothiazide (3) in the calibration solutions; **b** dihydralazine (8) and hydrochlorothiazide (5) in the presence of their degradation products (3, 4, 6, 7)

At the beginning, several trials were carried out to obtain good resolution between the drugs and their degradation products. They involved the use of different columns, mobile phases and flow rates. First, C8 and C18 columns (125 × 4.0 mm, 5 µm) were used and the mixtures containing water, methanol and acetonitrile as mobile phases. As far as C8 column was concerned, any mixture and any flow rate of the mobile phase (0.8–1.8 mL min^−1^) did not result in sufficient separation of hydrochlorothiazide and its degradation products. All peaks of interest showed retention times below 2 min. When a C18 column was used, it was showed that content of acetonitrile in the mobile phase could not exceed 20% to obtain symmetrical peaks of hydrochlorothiazide. However, its retention time was still very short and resolution between the peaks of degradation products was not achieved. Thus, addition of ion pair reagents was decided and one of them, i.e., tetrabutylammonium hydrogen sulfate was effective in good separation of the peaks of interest. However, retention of dihydralazine was too strong in these conditions (retention times above 20 min). Therefore, a CN column was tried together with mobile phases containing not more than 20% of acetonitrile. It was showed that the retention time of hydrochlorothiazide was still very short while that of dihydralazine was very long. Thus, acetonitrile content was decreased to 15% and ion pair reagent (tetrabutylammonium hydrogen sulphate) was added. As a result, the retention time of hydrochlorothiazide was extended to ca. 3 min, while that of dihydralazine shortened to ca. 13 min, maintaining an acceptable shape of the peaks. A huge difference in retention times of dihydralazine and hydrochlorothiazide was still observed but separation of all peaks of interest was possible. Respective chromatograms showed that the peaks of dihydralazine and hydrochlorothiazide were free from interferences of the degradation products, confirming selectivity of the method (Fig. 1).

Resistance to small changes in analytical parameters, i.e., acetonitrile content (15 ± 2%), pH (3.5 ± 0.1 unit), flow rate (1.4 ± 0.2 ml mL^−1^), detection wavelength (235 ± 3 nm) and column temperature (25 ± 2 °C) was examined. Uniformity of the obtained peak areas confirmed the robustness of the method. However, the calculated values of peak symmetry indicated sensitivity of the method to changes of detection wavelength and flow rate of the mobile phase.

The method was found to be linear over the concentration range of 20–120 µg mL^−1^ for both drugs, with average *R*^2^ of 0.9989 for dihydralazine and 0.9990 for hydrochlorothiazide. The calculated LOD and LOQ were 4.52 and 13.69 µg mL^−1^ for dihydralazine, and 0.90 and 2.74 µg mL^−1^ for hydrochlorothiazide. The RSD values in the range 0.59–1.16% for dihydralazine and 0.47–1.72% for hydrochlorothiazide (1-day precision), and 0.90–1.07% for dihydralazine and 0.81–1.57% for hydrochlorothiazide (inter-day precision) were obtained. Accuracy of the method was confirmed by determining both drugs in powdered tablets. Recovery values were obtained in the range 99.24–100.99% for dihydralazine and 99.45–101.54% for hydrochlorothiazide (Table 1). The chromatograms obtained for the samples of powdered tablets showed the peaks of interest free from interferences of excipients, confirming selectivity of the method.

**Table 1:** Validation of HPLC-UV method for simultaneous determination of dihydralazine and hydrochlorothiazide

Parameter	Dihydralazine	Hydrochlorothiazide
Linearity range (µg mL^−1^)	20–120	20–120
Slope	344,398	271,311
SD of slope	3134	1361
Intercept	− 2,333,333	− 127,834
SD for intercept	471,404	74,342
*R* ^2^	0.99886	0.99895
SD of *R*^2^	0.00053	0.00032
LOD (µg mL^−1^)	4.52	0.90
LOQ (µg mL^−1^)	13.69	2.74
Accuracy (% recovery)	99.24–101.03	99.45–101.54
Precision (RSD)		
Intra-day	0.59–1.16	0.47–1.72
Inter-day	0.90–1.07	0.81–1.57
Retention time (min)	13.21	2.73
Asymmetry factor	1.5	1.0

### Degradation in a Solid State

Our study showed that dihydralazine was sensitive to high temperature and humidity (22.01% degradation after 2 months at 70 °C/80% RH). In the mixture with hydrochlorothiazide, there was further increase of degradation to 29.70% (Table 2). Therefore, it could be concluded that degradation of dihydralazine was accelerated by hydrochlorothiazide. In the literature, a similar effect of hydrochlorothiazide was documented for its solid mixture with cilazapril [38].

**Table 2:** Percentage level of degradation of dihydralazine and hydrochlorothiazide under high temperature/humidity and under UV/Vis light

Stress conditions	Level of degradation (%)			
	Dihydralazine		Hydrochlorothiazide	
	Individual	Mixture	Individual	Mixture
70 °C/80% RH	22.00	29.70	12.83	17.72
1 ICH	2.85	28.98	16.71	19.93
3 ICH	13.26	59.58	27.78	33.99
6 ICH	100.0	100.0	66.92	89.42

1 ICH = 18,902 kJ m^2−1^, 3 ICH = 56,706 kJ m^2−1^; 6 ICH = 113,412 kJ m^2−1^

After 2 months at 70 °C/80% RH, hydrochlorothiazide as individual showed degradation equal to 12.83%. According to the literature, the highest degradation of hydrochlorothiazide (ca. 15%) occurred at 100 °C (after 5 h) [25] and 65 °C (after 24 h) [26]. In the mixture with dihydralazine, an increase of hydrochlorothiazide degradation to 17.72% was observed (Table 2).

These data allowed to draw the conclusion on mutual influence of both dihydralazine and hydrochlorothiazide on their stability. As a consequence, the need to adequately protect these active substances from high temperature and humidity was clearly shown.

### Photodegradation

**Fig. 2:**
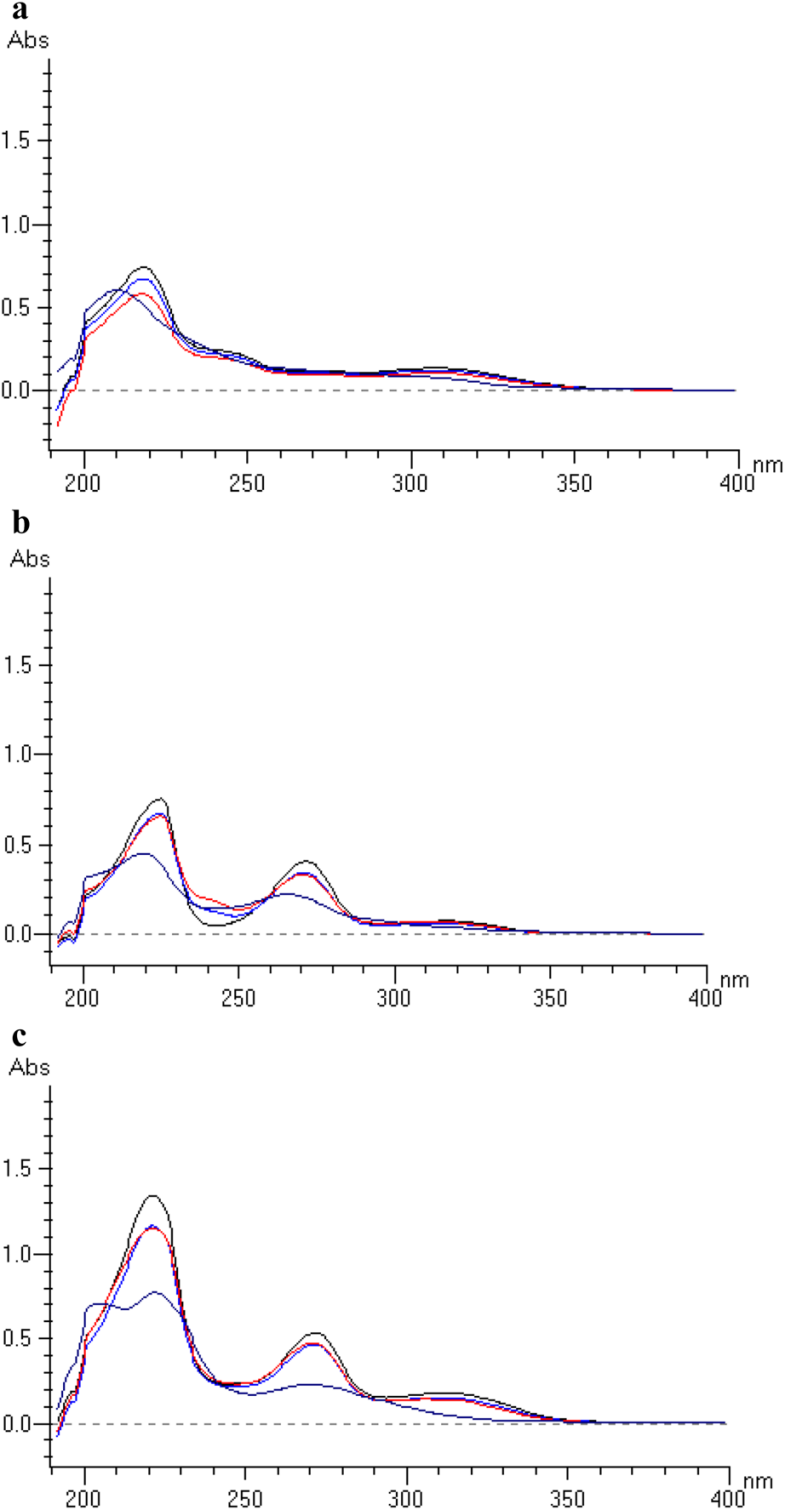
**a** UV/Vis spectra of dihydralazine, **b** hydrochlorothiazide and **c** their mixture after photodegradation study: black line—standard (non-stressed sample), blue line—sample under 1 ICH dose, red line—sample under 3 ICH doses and violet line—sample after 6 ICH doses

In our study, the energy of 18,902 kJ m^2−1^ or 1 ICH dose was equivalent to 1,200,000 lx h and 200 W m^2−1^ [39]. Dihydralazine fulfilled the requirements of the test confirming photostability, because its percentage degradation was 2.85%. However, under higher exposures, its degradation was 13.26% (56,706 kJ m^2−1^ or 3 ICH doses) and 100% (113,412 kJ m^2−1^ or 6 ICH doses). In addition, its UV/Vis spectrum changed after these expositions (Fig. 2a). Dihydralazine mixed with hydrochlorothiazide underwent higher photodegradation than an individual substance. Under energy equal 1 ICH dose, the level of degradation rose to 28.98% (Table 2). These data allowed to draw the conclusion that hydrochlorothiazide increased sensitivity of dihydralazine to light. It was also observed as changes in respective UV/Vis spectra (Fig. 2c). To the best of our knowledge, the results presented here are the first in the area of photostability of dihydralazine.

Present experiment showed that hydrochlorothiazide was sensitive to light (16.71% degradation upon energy equivalent to 1 ICH dose) (Table 2). According to the literature, similar level of degradation (ca. 18%) was observed under monochromatic light at 256 nm [25]. However, much lower photodegradation (0.1–5.5%) was also reported [29, 30]. In our experiment, the influence of light was confirmed by changes in the UV/Vis spectra of the stressed samples. After 6 ICH doses, a clear shifting of one of the absorption maximum of hydrochlorothiazide was observed (Fig. 1b). It confirmed photosensitivity of hydrochlorothiazide and indicated the need to apply appropriate photoprotection for pure drug and its formulations.

Hydrochlorothiazide mixed with dihydralazine was more degraded in comparison with an individual substance (19.93 vs. 16.71%, 33.99 vs. 27.78% and 89.42 vs. 66.92% after 1, 3 and 6 ICH doses, respectively) (Table 2). The above data as well as respective UV/Vis spectra confirmed the mutual influence of both components on their photostability (Fig. 2c).

### Degradation in a Liquid State

The present study proved stability of dihydralazine in a strongly acidic environment (1.61% of degradation in 1 M HCl after 300 min). On the contrary, the highest decomposition was observed in a strongly alkaline environment (100% of degradation in 1 M NaOH). In addition, the substance degraded in buffers of pH 4, 7 and 10 (5.50, 38.54 and 74.98% of degradation, respectively). Dihydralazine in a mixture was degraded to a much greater extent. The greatest increase of degradation occurred in the buffer of pH 7 (100 vs. 38.54%). In addition, a high level of degradation (51.06%) was observed in 1 M HCl, where individually stressed dihydralazine showed stability (Table 3).

**Table 3:** Percentage level of degradation and kinetic parameters of degradation of dihydralazine in solutions

Stress conditions	Degradation after 300 min (%)	Linear equation, *y* = *ax* + *b*	*R* ^2^	*k* (s^−1^)	*t*_0.5_ (h)
Dihydralazine					
1 M HCl	1.61	nc	nc	nc	nc
Buffer pH 4	5.50	nc	nc	nc	nc
Buffer pH 7	38.54	*y* = − 0.0018*x* + 4.7378	0.8820	2.70 × 10^−5^	7.13
Buffer pH 10	74.98	*y* = − 0.0043*x* + 4.7449	0.9665	7.70 × 10^− 5^	2.50
1 M NaOH	100.0	*y* = − 0.0184*x* + 4.0556	0.9989	5.09 × 10^−4^	0.38
Dihydralazine in the mixture with hydrochlorothiazide					
1 M HCl	51.06	*y* = − 0.0064*x* + 4.9381	0.9871	3.97 × 10^−5^	4.85
Buffer pH 4	82.39	*y* = − 0.0064*x* + 4.9381	0.9871	9.65 × 10^−5^	1.99
Buffer pH 7	100.0	*y* = − 0.0080*x* + 4.4918	0.8243	3.28 × 10^−4^	1.50
Buffer pH 10	100.0	*y* = − 0.0092*x* + 4.7455	0.9805	3.53 × 10^−4^	1.26
1 M NaOH	100.0	*y* = − 0.0047*x* + 4.6211	0.9866	9.02 × 10^−5^	2.13

*nc* non-calculated

According to the cited works, the highest degradation of hydrochlorothiazide occurred in methanol (53.56%) [25], 1 M NaOH (69.60%) and 1 M HCl (81.70%) [21]. In other works, degradation in acidic medium ranged from 0.4 to 17.86% while in alkaline medium from 0.15 to 36.82% [19, 20, 22, 25, 28, 30–32]. Hydrochlorothiazide was shown to be stable in the buffer of pH 2.0 and less stable at pH 9.0, where 9.07% of the drug degraded [23]. Our experiments confirmed that hydrochlorothiazide was prone to degradation in 1 M HCl (52.29%) and 1 M NaOH (37.97%). Degradation in buffers of pH 4, 7 and 10 was higher in comparison to Ref. 23 (23.64–36.99%), probably because of using higher temperature. Degradation of hydrochlorothiazide was further intensified in the mixture with dihydralazine. The greatest increase of degradation occurred in the buffer of pH 7 (93.72 vs. 36.34%) (Table 4).

**Table 4:** Percentage level of degradation and kinetic parameters of degradation of hydrochlorothiazide in solutions

Stress conditions	Degradation after 300 min (%)	Linear equation, *y* = *ax* + *b*	*R* ^2^	*k* (s^−1^)	*t*_0.5_ (h)
Hydrochlorothiazide					
1 M HCl	52.29	*y* = − 0.0018*x* + 4.5315	0.8117	3.11 × 10^−5^	4.68
Buffer pH 4	23.64	*y* = − 0.0007*x* + 4.7441	0.9366	1.50 × 10^−5^	12.83
Buffer pH 7	36.34	*y* = − 0.0011*x* + 4.6498	0.9642	2.50 × 10^−5^	7.67
Buffer pH 10	36.99	*y* = − 0.0012*x* + 4.7004	0.9506	2.51 × 10^−5^	7.49
1 M NaOH	37.97	*y* = − 0.0006*x* + 4.7518	0.9781	1.29 × 10^−5^	15.91
Hydrochlorothiazide in the mixture with dihydralazine					
1 M HCl	53.98	*y* = − 0.0019*x* + 4.5717	0.9862	4.31 × 10^−5^	4.47
Buffer pH 4	70.71	*y* = − 0.0038*x* + 4.5422	0.9391	6.79 × 10^−5^	2.82
Buffer pH 7	93.72	*y* = − 0.0096*x* + 4.4038	0.9143	1.54 × 10^−4^	1.25
Buffer pH 10	94.14	*y* = − 0.0081*x* + 4.6094	0.9190	1.58 × 10^−4^	1.22
1 M NaOH	47.56	*y* = − 0.0017*x* + 4.7004	0.9438	3.59 × 10^−5^	5.36

#### Kinetics

**Fig. 3:**
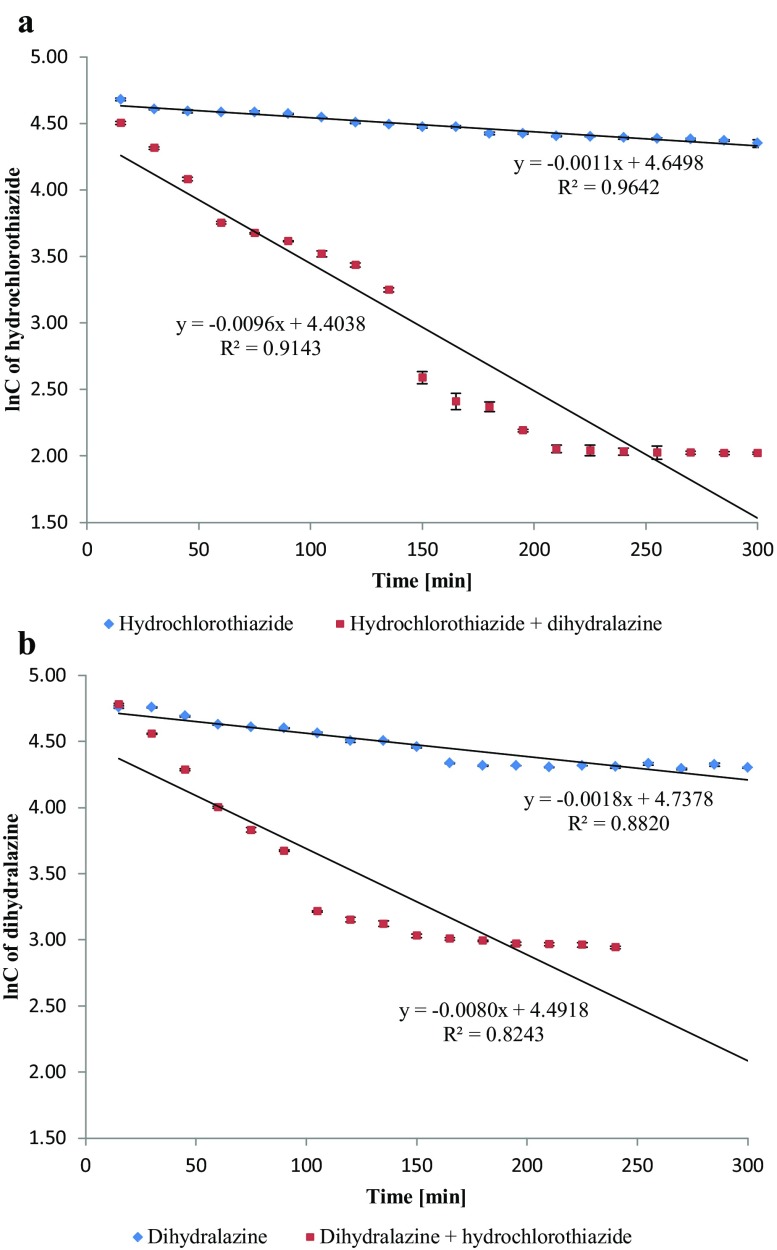
First-order plots of drugs degradation in the buffer of pH 7: **a** dihydralazine degraded as an individual and in the mixture with hydrochlorothiazide; **b** hydrochlorothiazide degraded as an individual and in the mixture with dihydralazine. Errors bars on the curves represent the SD values of triplicate samples

We completed our experiments by calculating the degradation kinetics of dihydralazine and hydrochlorothiazide. It was found that degradation of dihydralazine, individually stressed and in the mixture, proceeded as the first-order reactions. Values of the rate constants (*k*) were at the levels of 10^−5^–10^−4^ s^−1^. The shortest *t*_0.5_ (0.38 h) was calculated for 1M NaOH (Table 3). Hydrochlorothiazide degradation was also observed as the first-order reactions. Values of the observed *k* for individually stressed hydrochlorothiazide were at the level of 10^−5^ s^−1^. The shortest *t*_0.5_ was obtained in 1 M HCl (4.68 h). In the mixture, the quickest degradation of hydrochlorothiazide was calculated at pH 7 and 10 (*k* values at the level 10^−4^ s^−1^), where the lowest values of *t*_0.5_ were obtained (1.22–1.25 h) (Table 4). In the mixture, both the substances were observed to degrade harder and faster in almost all experimental conditions. However, the biggest differences occurred in the buffer of pH 7. Therefore, respective data were depicted as *xy* diagrams (Fig. 3). Due to the lack of other data in this area, the results presented here are a valuable supplement to the literature resources.

### Identification of Degradation Products

**Fig. 4:**
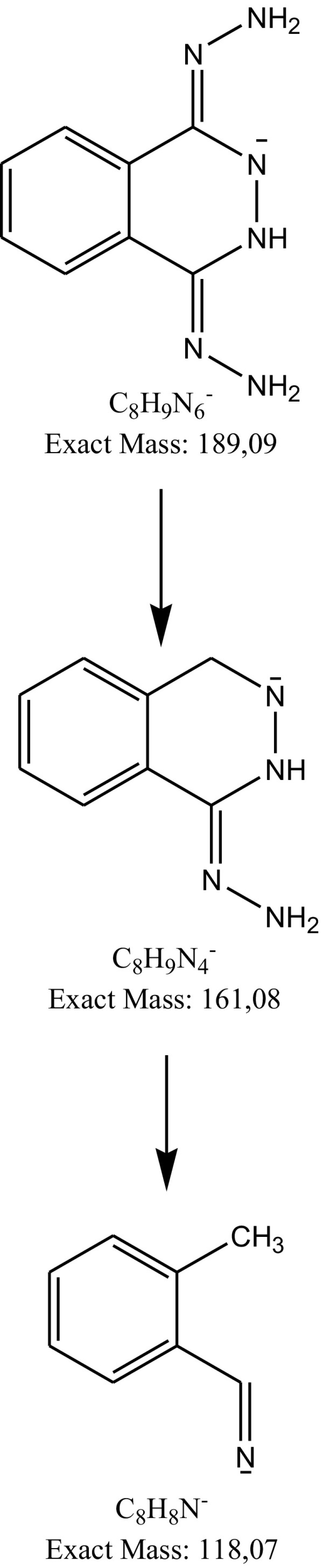
Fragmentation pattern of dihydralazine in negative ionization mode: CID off set 10V

**Fig. 5:**
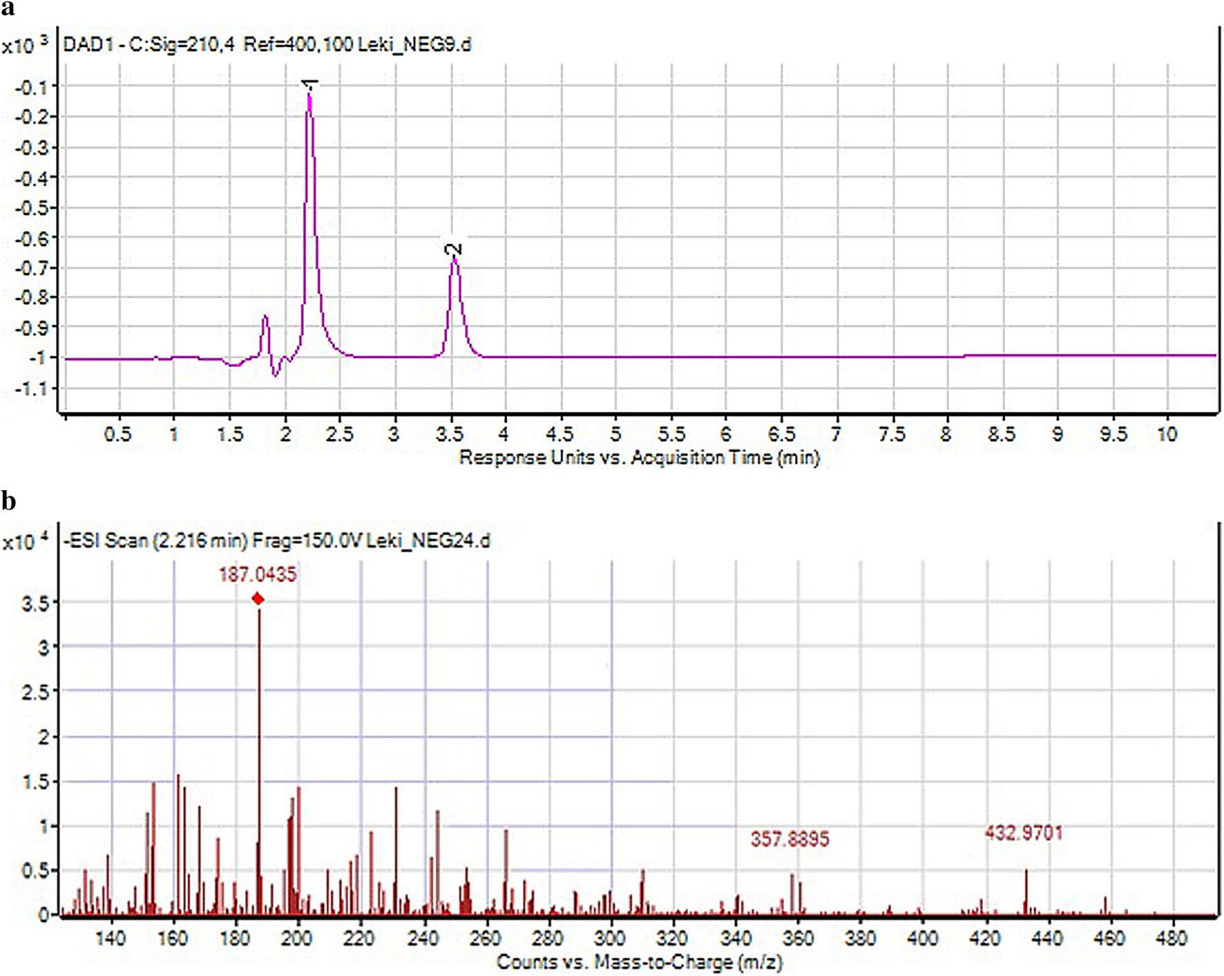
**a** Chromatogram DAD of dihydralazine (1) and its degradation product (2) in 1M NaOH. **b** Negative ion ESI LC/MS of degradation product of dihydralazine (1,4-dihydrazinylidenophthalazine); CID off set 10 V

**Fig. 6:**
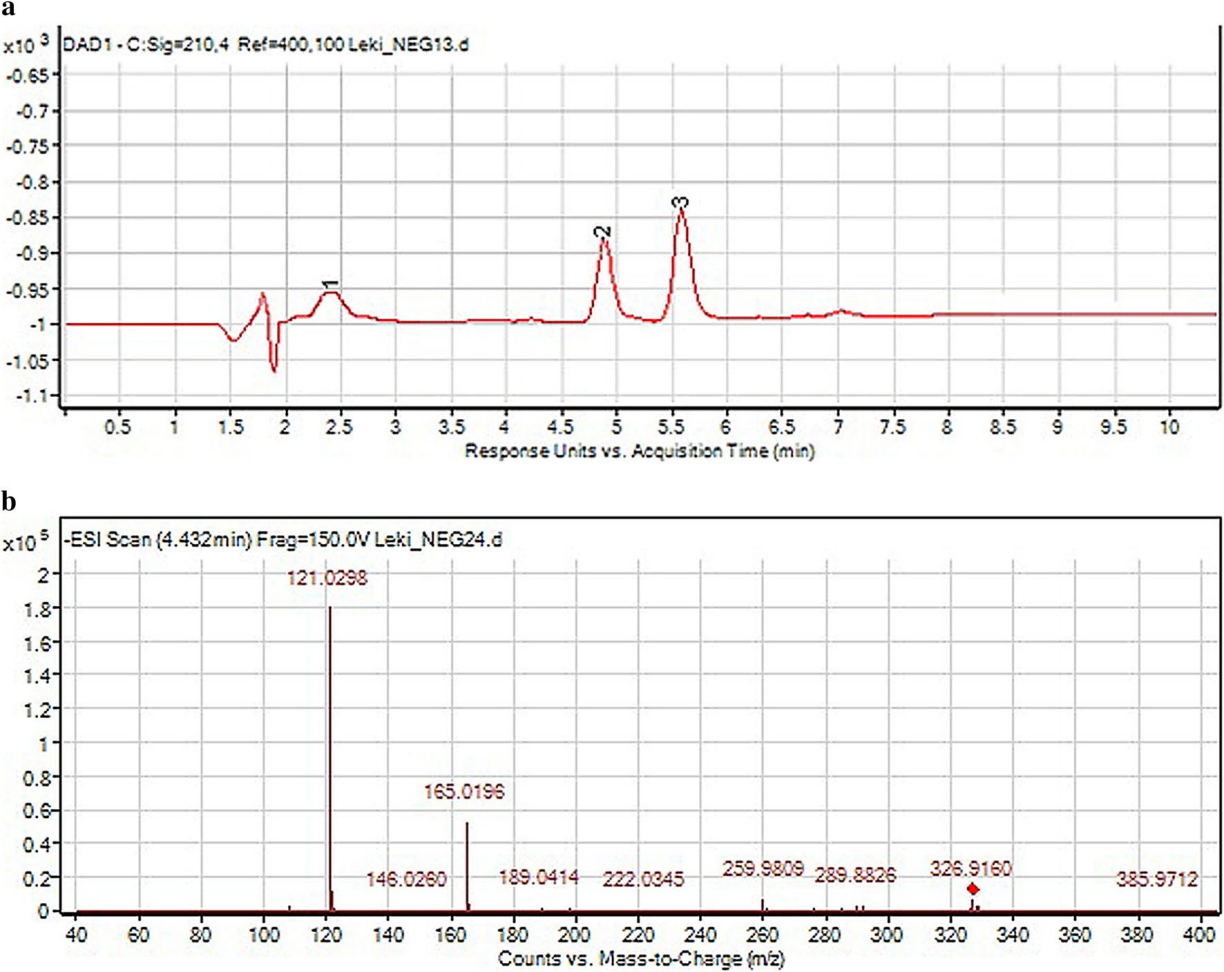
**a** Chromatogram DAD of dihydralazine (1) and its degradation products (2, 3) under UV/Vis light. **b** Negative ion ESI LC/MS of degradation product of dihydralazine (2): CID of set 10 V

**Fig. 7:**
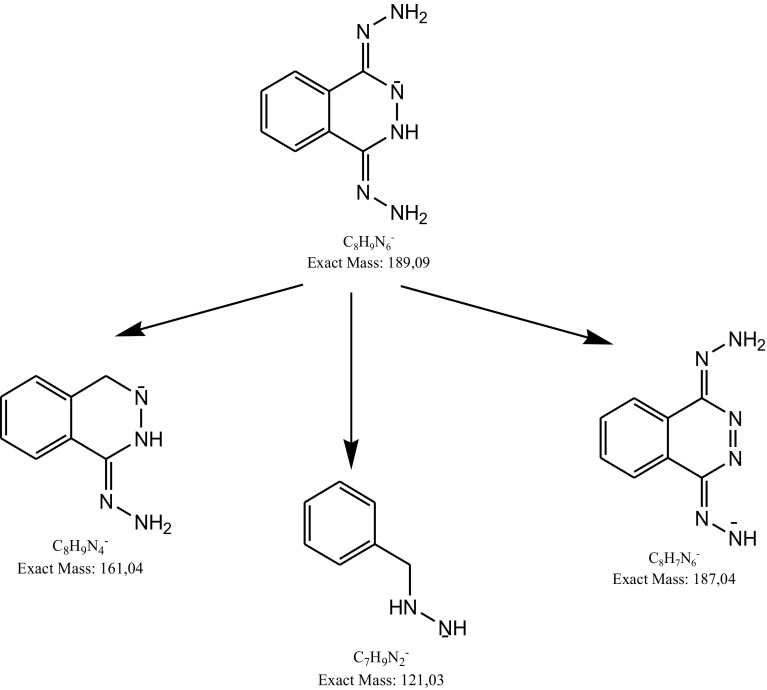
Proposed degradation pathway of dihydralazine in 1 M NaOH and under UV/Vis light

As a result of negative ionization of dihydralazine, a deprotonated molecule [M–H]^−^ of *m*/*z* 189.09 was formed. Then, fragment ions of *m*/*z* 161.08 and 118.07 were produced. Based on these results, it was shown that dihydralazine was fragmented by loss of hydrazine and breaking the phenazine ring (Fig. 4). The Eur. Ph. monograph of dihydralazine lists three impurities, A (4-hydrazinophthalazin-1-amine), B (hydrazine) and C (1-hydrazinophthalazine) [2]. In our LC-DAD and LC/MS studies, a product of *m*/*z* 187.04 was observed after degradation in 1 M NaOH. The respective chromatogram and mass spectrum were presented in Fig. 5. It was identified as 1,4-dihydrazinylidenophthalazine (Table 5). Such a product was not described in the literature so far. In the buffer of pH 10 and under UV/Vis light, two degradation products were observed as fragment ions of *m*/*z* 121.03 and 161.04 (Fig. 6). The first product was not described in the literature so far, while the second one was identified as impurity C (Table 5). The proposed degradation pathways for dihydralazine are presented in Fig. 7.

**Table 5:**
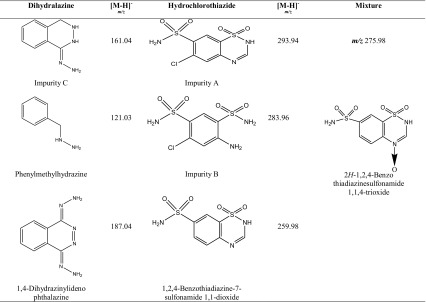
The identified degradation products of dihydralazine and hydrochlorothiazide

**Fig. 8:**
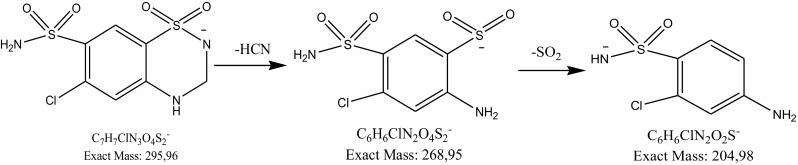
Fragmentation pattern of hydrochlorothiazide in negative ionization mode: CID off set 10 V

**Fig. 9:**
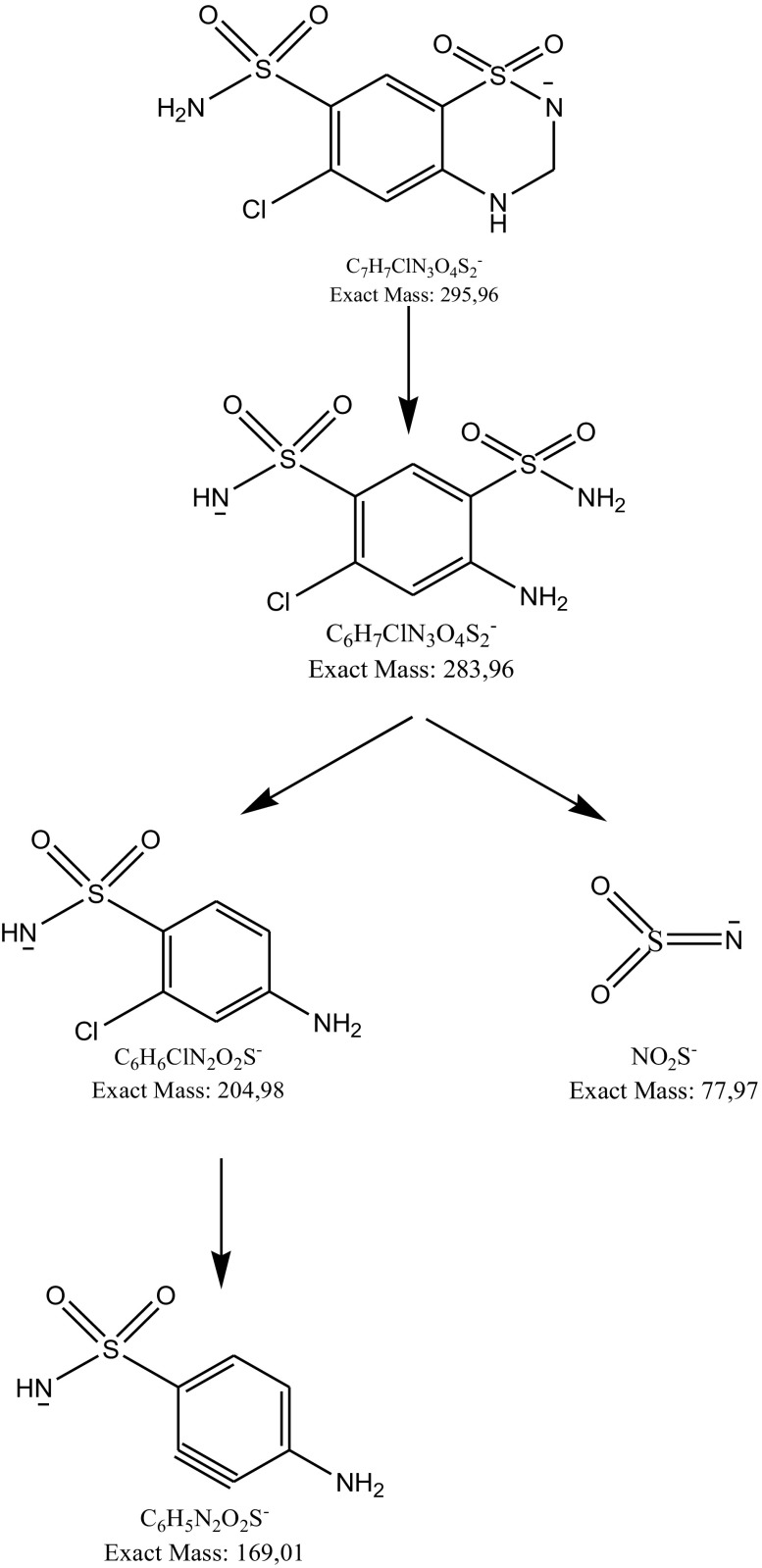
Proposed degradation pathway of hydrochlorothiazide in solutions

Using a negative ionization mode, a deprotonated molecule [M–H]^−^ of hydrochlorothiazide of *m*/*z* 295.06 was obtained from which, after losing the HCN molecule, an ion of *m*/*z* 268.95 was formed. Further fragmentation led to formation of an ion of *m*/*z* 204.98 (loss of SO_2_) (Fig. 8). These results are consistent with previous results concerning fragmentation of hydrochlorothiazide [27]. The Eur. Ph. monograph of hydrochlorothiazide lists three impurities, A (6-chloro-2H-1,2,4-benzothiadiazine-7-sulfonamide 1,1-dioxide, chlorothiazide), B (4-amino-6-chlorobenzene-1,3-disulfonamide) and C (6-chloro-*N*-[(6-chloro-7-sulfamoyl-2,3-dihydro-4H-1,2,4-benzothiadiazin-4-yl 1,1-dioxide)methyl]-3,4-dihydro-2H-1,2,4-benzothiadiazine-7-sulfonamide 1,1-dioxide) [2]. In the present study, a degradation product of hydrochlorothiazide was detected as a fragment ion of *m*/*z* 283.96. It was identified as impurity B. It confirmed the results obtained by Belal et al. [22] and Mahajan et al. [27] where the same product was detected as a result of acid and alkaline hydrolysis of hydrochlorothiazide. The structure of the resulting product indicated breaking of the 1,2,4-benzothiadiazine ring and loss of formaldehyde. The resulting product further fragmented yielding an ion of *m*/*z* 204.98 (loss of the sulfonamide group), from which hydrogen chloride was detached to form an ion of* m*/*z* 169.9. The proposed fragmentation profile was drawn in Fig. 9.

**Fig. 10:**
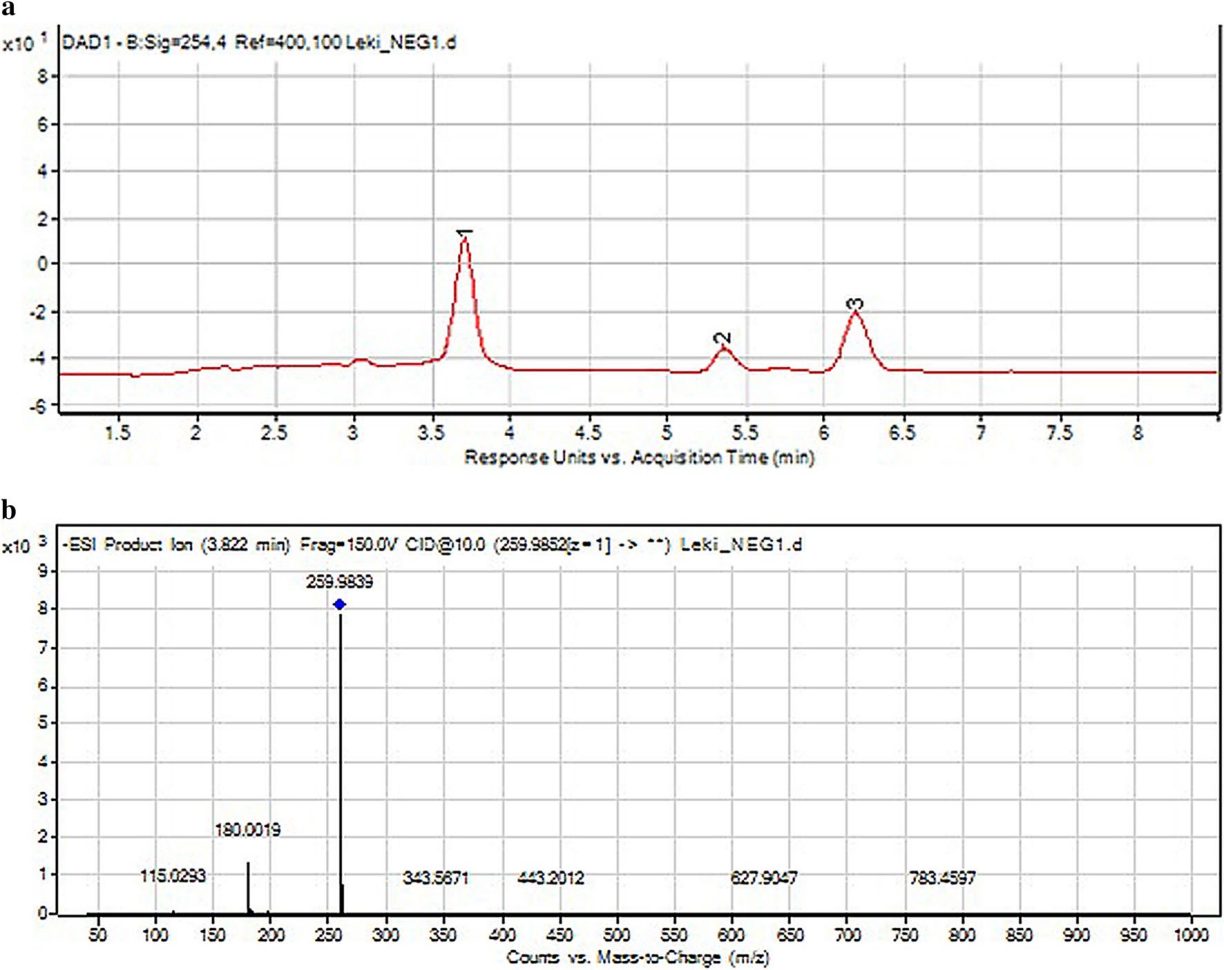
**a** Chromatogram DAD of hydrochlorothiazide (3) and its degradation products (1, 2) under UV/Vis light. **b** Negative ion ESI LC/MS of degradation product of hydrochlorothiazide (1): CID off set 10V

**Fig. 11:**
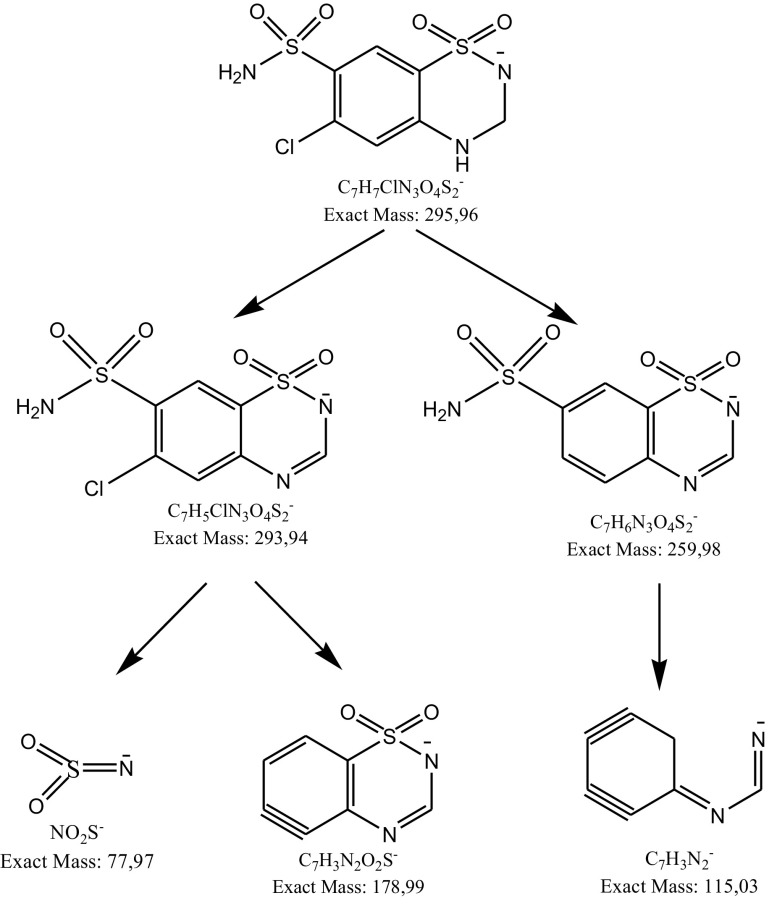
Proposed degradation pathway of hydrochlorothiazide under UV/Vis light

Under UV/Vis light, two other fragment ions of *m*/*z* values 293.94 and 259.98 were detected (Fig. 10). The first was identified as chlorothiazide (impurity A) [2]. The second product was not described in the literature so far and was identified as 1,2,4-benzothiadiazine-7-sulfonamide 1,1-dioxide (Table 5). Based on these results, it was concluded that light facilitated detaching the chlorine atom from the aromatic ring (Fig. 11). A similar result was observed and described for amiloride [40]. In the present experiment, chlorothiazide further fragmented by losing the sulfonamide group and hydrogen chloride yielding an ion of *m*/*z* 178.99. Previously, only chlorothiazide was described as a result of photodegradation of the parent drug [27].

**Fig. 12:**
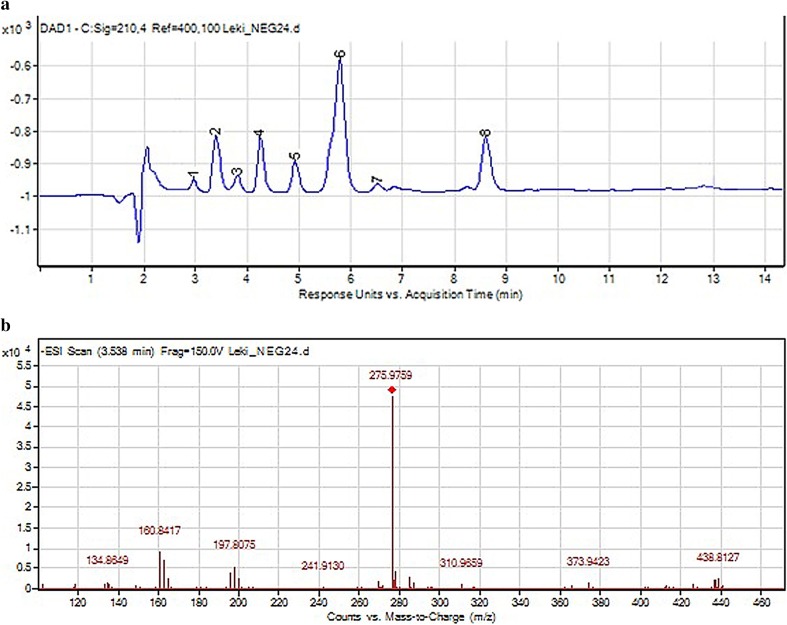
**a** Chromatogram DAD of hydrochlorothiazide (7) and degradation products of hydrochlorothiazide and dihydralazine (1–6, 8) in the mixture under UV/Vis light. **b** Negative ion ESI LC/MS of degradation product of hydrochlorothiazide (2): CID off set 10 V

**Fig. 13:**
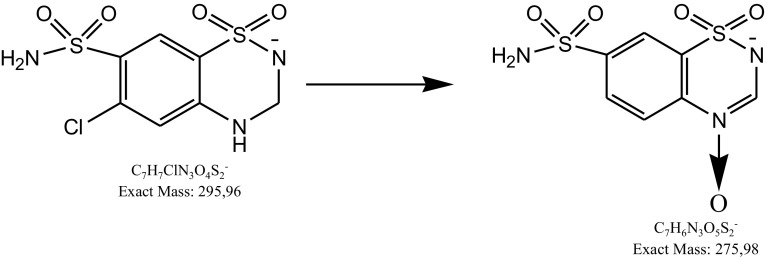
Proposed photodegradation pathway of hydrochlorothiazide in presence of dihydralazine

Most of these degradation products were observed in the mixture of dihydralazine and hydrochlorothiazide (fragment ions of *m*/*z* 121.03, 169.09, 178.99, 187.04, 204.98 and 293.94). In addition, further products occurred after degradation in 1 M HCl (*m*/*z* 199.07), 1 M NaOH, buffers (*m*/*z* 211.07) and under UV/Vis light (*m*/*z* 169.05). Unfortunately, they were not identified by us. However, quite a new product of photodegradation of hydrochlorothiazide of *m*/*z* 275.98 was detected in the mixture (Fig. 12) and identified as 2*H*-1,2,4-benzothiadiazinesulfonamide 1,1,4-trioxide (Table 5). This indicated a new way of hydrochlorothiazide photodegradation by detaching the chlorine atom and oxidation at the nitrogen atom (Fig. 13).

## Conclusions

Dihydralazine in a solid state was sensitive to high temperature and humidity, while in solutions it was degraded by UV/Vis light and pH below 4. Hydrochlorothiazide in a solid state was sensitive to high temperature and humidity as well as to UV/Vis light. In solutions, it degraded in 1M HCl, 1M NaOH, buffers and under UV/Vis light. In the stressed individual samples of dihydralazine and hydrochlorothiazide, (phenylmethyl)hydrazine and 1,2,4-benzothiadiazine-7-sulfonamide 1,1-dioxide were observed for the first time.

Percentage degradation of drugs in the mixture was greater than of individual drugs under the same stress conditions. An interesting effect was observed for dihydralazine which became sensitive to acidic degradation. Increased sensitivity of both active substances to stress and potent interactions between them were confirmed by new degradation products detected, e.g., 2H-1,2,4-benzothiadiazine-7-sulfonamide 1,1,4-trioxide.

The results presented here complemented the current knowledge about degradation processes of dihydralazine and hydrochlorothiazide. These data may be the starting point for further studies on new degradation products in terms of their potential toxicity and then, for qualifying them as new related substances in pharmacopoeial monographs. These data may also serve as a starting point for designing new two-component formulations.
